# Supplementary material for: Causal Relationship between Aging and Anorexia Nervosa: A White-Matter-Microstructure-Mediated Mendelian Randomization Analysis
Source: Biomedicines. 2024 Aug 16;12(8):1874. doi: 10.3390/biomedicines12081874 (PMC11351342; doi:10.3390/biomedicines12081874)
Supplement: Supplementary file 1 [file biomedicines-12-01874-s001.zip › Supplementary Figure/Figure S7/Single SNP plot of MR analysis for ubm-b-1470 to AN.pdf]

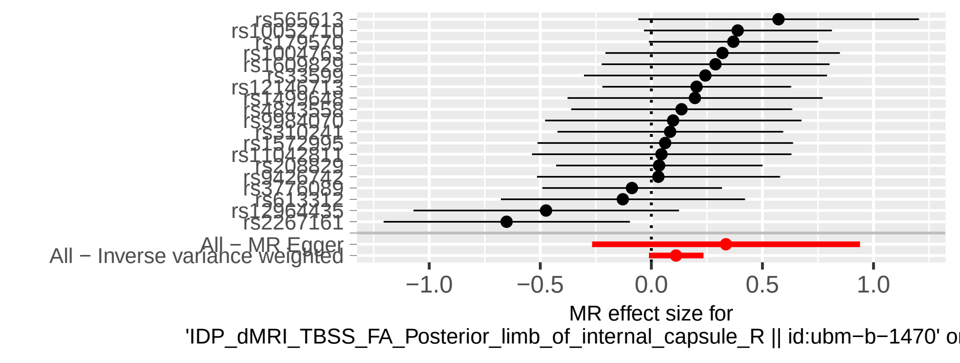
rs565613
rs10052710
rs179570
rs1004763
rs1609829
rs33599
rs12146713
rs1499648
rs4843558
rs9984070
rs310241
rs1572995
rs11042811
rs208829
rs9426742
rs3776089
rs613312
rs12964435
rs2267161
All − MR Egger
All − Inverse variance weighted
−1.0
−0.5
0.0
0.5
1.0
MR effect size for
'IDP_dMRI_TBSS_FA_Posterior_limb_of_internal_capsule_R || id:ubm−b−1470' or
